# Translation of a Leaderless Reporter Is Robust During Exponential Growth and Well Sustained During Stress Conditions in *Mycobacterium tuberculosis*

**DOI:** 10.3389/fmicb.2021.746320

**Published:** 2021-09-17

**Authors:** Anna D. Grabowska, Nuria Andreu, Teresa Cortes

**Affiliations:** Department of Infection Biology, Faculty of Infectious and Tropical Diseases, London School of Hygiene and Tropical Medicine, London, United Kingdom

**Keywords:** *Mycobacterium tuberculosis*, translation, leaderless transcript, stress conditions, Shine–Dalgarno sequence, reporter strains, bioluminescence

## Abstract

*Mycobacterium tuberculosis* expresses a large number of leaderless mRNA transcripts; these lack the 5′ leader region, which usually contains the Shine–Dalgarno sequence required for translation initiation in bacteria. In *M. tuberculosis*, transcripts encoding proteins like toxin–antitoxin systems are predominantly leaderless and the overall ratio of leaderless to Shine–Dalgarno transcripts significantly increases during growth arrest, suggesting that leaderless translation might be important during persistence in the host. However, whether these two types of transcripts are translated with differing efficiencies during optimal growth conditions and during stress conditions that induce growth arrest, is unclear. Here, we have used the *desA1* (Rv0824c) and *desA2* (Rv1094) gene pair as representative for Shine–Dalgarno and leaderless transcripts in *M. tuberculosis* respectively; and used them to construct bioluminescent reporter strains. We detect robust leaderless translation during exponential *in vitro* growth, and we show that leaderless translation is more stable than Shine–Dalgarno translation during adaptation to stress conditions. These changes are independent from transcription, as transcription levels did not significantly change following quantitative real-time PCR analysis. Upon entrance into nutrient starvation and after nitric oxide exposure, leaderless translation is significantly less affected by the stress than Shine–Dalgarno translation. Similarly, during the early stages of infection of macrophages, the levels of leaderless translation are transiently more stable than those of Shine–Dalgarno translation. These results suggest that leaderless translation may offer an advantage in the physiology of *M. tuberculosis*. Identification of the molecular mechanisms underlying this translational regulation may provide insights into persistent infection.

## Introduction

Tuberculosis, caused by *Mycobacterium tuberculosis*, is mainly a pulmonary disease and the leading cause of death worldwide from a single bacterial infectious agent, with nearly 1.5 million deaths each year (WHO, 2020). In 2019, world tuberculosis incidence exceeded 10 million people (WHO, 2020), and it is estimated that a quarter (1.7 billion individuals) of the human population is latently infected with *M. tuberculosis* (Houben and Dodd, 2016). This reflects the complex life cycle of this pathogen, that can involve prolonged periods of asymptomatic infection, where the bacteria enter a non-replicating state, prior to the onset of disease. During this process, bacteria have to adapt to diverse stresses, including nutrient and oxygen limitation, and exposure to reactive oxygen and nitrogen species within host cells (Forrellad et al., 2013), primarily alveolar macrophages. The downregulation of key life processes, like transcription and translation, has been associated with *M. tuberculosis* tolerance to stresses, usually via non-replicating persistence (Bentrup and Russell, 2001; Ehrt et al., 2018).

Biological adaptation mediated by translation regulation has been increasingly associated to changes in ribosomal composition, both in bacteria and eukaryotes (Xue and Barna, 2012; Byrgazov et al., 2013). These changes in ribosomal composition can result in the selective translation of different types of mRNA transcripts. In bacteria, the canonical transcript structure includes a 5′ untranslated region (5′ UTR) that harbors important regulatory sequences like the Shine–Dalgarno sequence required for translation initiation. Canonical translation of bacterial transcripts is initiated by binding of the Shine–Dalgarno sequence to the complementary region of 16S ribosomal RNA in the 30S small subunit of the ribosome (Shine and Dalgarno, 1974). But bacterial transcripts that completely lack a 5′UTR and hence the Shine–Dalgarno sequence, known as leaderless transcripts, also exist (Zheng et al., 2011; Nakagawa et al., 2017). The absence of a Shine–Dalgarno sequence has important implications for the initiation of translation. In *Escherichia coli*, leaderless transcripts are translated with low efficiency when a 70S ribosome directly binds to the ATG start codon (Udagawa et al., 2004; Beck et al., 2016). This preference can be altered by antibiotics or toxin-antitoxin systems, that generate subpopulations of specialized ribosomes that selectively translate leaderless transcripts (Kaberdina et al., 2009; Vesper et al., 2011). Previous research in *M. tuberculosis* has shown that approximately 25% of its mRNA transcripts are expressed as leaderless transcripts (Cortes et al., 2013; Shell et al., 2015), a substantially higher percentage than that reported in other bacterial pathogens (1.2–3%) (Sharma et al., 2010; Kröger et al., 2012; Seo et al., 2012; Thomason et al., 2015). In the model organism *Mycobacterium smegmatis*, which also contains a similar percentage of leaderless transcripts to that of *M. tuberculosis*, comparable translation rates for canonical and leaderless transcripts have been reported (Shell et al., 2015; Nguyen et al., 2020). Altogether, this suggests that leaderless translation might have a more central role in the regulation of mycobacterial physiology than in that of *E. coli*. Indeed, proteins with secondary adaptive functions, like toxin–antitoxin systems, are generally leaderless in *M. tuberculosis* and the overall ratio of leaderless to canonical Shine–Dalgarno transcripts increases during incubation in a starvation model of growth arrest (Cortes et al., 2013). These observations suggest that the regulation of translation of Shine–Dalgarno and leaderless transcripts might differ during different stresses, such as during nutrient deprivation and macrophage infection; however, this has not been investigated.

Here we aimed to better understand the role of leaderless translation in the response of *M. tuberculosis* to *in vitro* stress and during infection. To this end, we quantified translation differences between leaderless and Shine–Dalgarno transcripts during different growth conditions and during macrophage infection using luminescent *M. tuberculosis* strains harboring leaderless and Shine–Dalgarno reporter constructs. Quantification of luminescence levels during exponential growth and during nutrient starvation, exposure to nitric oxide (NO) and infection of macrophages revealed robust and sustained leaderless translation in *M. tuberculosis*. Our results suggest that leaderless translation may offer an advantage to Shine–Dalgarno translation during adaptation to different growth conditions.

## Materials and Methods

### Bacterial Strains and Growth Conditions

*Mycobacterium tuberculosis* H37Rv (SysteMTb), *Mycobacterium smegmatis* mc2155 (Snapper et al., 1990) and *Escherichia coli* NEB-5α and NEB-10β (New England Biolabs UK Ltd) were used in this study. All the strains were grown at 37°C, either in a shaking incubator (*E. coli* and *M. smegmatis*) or in a roller apparatus (*M. tuberculosis)*. All work involving live *M. tuberculosis* was performed in a dedicated Biosafety Level 3 (BSL3) laboratory. *M. tuberculosis* and *M. smegmatis* were grown on Middlebrook 7H11 agar medium (BD Diagnostics) supplemented with 0.5% glycerol and 10% oleic acid albumin-dextrose-catalase (OADC) enrichment (BD Diagnostics). Liquid cultures of mycobacteria were grown in Middlebrook 7H9 broth (BD Diagnostics) supplemented with 0.2% glycerol, 10% albumin-dextrose-catalase (ADC) enrichment (BD Diagnostics) and 0.05% Tween 80 (Sigma). For the nutrient starvation and nitric oxide expression screens, *M. tuberculosis* was cultured in 100 ml of liquid medium in 1 L roller bottles rolling at 2 rpm, unless stated otherwise. *E. coli* was cultured on Luria-Bertani (LB) agar and in LB liquid medium. Kanamycin was added where appropriate [25 μg ml^–1^ for mycobacteria, 50 μg ml^–1^ for *E. coli* (Sigma)]. The exponential and stationary phases of growth were defined based on the growth curves obtained. For *E. coli*, exponential growth was considered for time points from 2 to 5 h and stationary growth from 6 to 9 h. For *M. smegmatis*, the exponential phase of growth was defined for time points from 6 to 12 h and stationary for time points 24 to 48 h. Finally, for *M. tuberculosis*, exponential growth phase was considered for time points 2–8 days and non-exponential growth from 11 days, which corresponded to 1 week after OD_600_ had reached 1.0.

### DNA Manipulations

Mycobacterial genomic DNA was prepared using the InstaGene Matrix kit (BioRad) or the PureLink Genomic DNA Kit (Invitrogen), following manufacturers’ instructions. All the oligonucleotides used in this study are listed in Table 1 and were synthesized by Sigma-Aldrich or IDT. DNA sequences were determined by Source BioScience^1^ using the Sanger method.

**TABLE 1:** Primers used in this study.

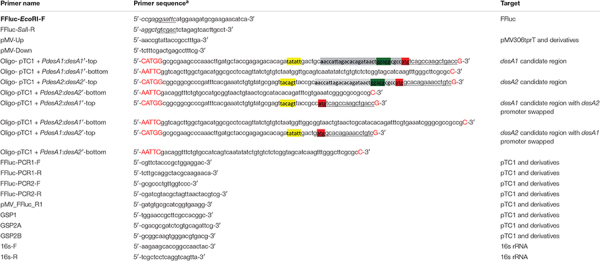

*^*a*^In italics, sequence added to include restriction sites (underlined) for cloning procedures. For the DNA oligos, in capitals red, bases added to complement the overhangs generated during cloning procedures; in highlighted yellow, –10 promoter motif; in bold, transcription start site as described in Cortes et al. (2013) highlighted gray, 5′UTR sequence; in highlighted green, Shine–Dalgarno sequence; underlined, sequence corresponding to the six N-terminal amino acids with the start codon highlighted in red.*

### Construction of Luminescent Reporter Plasmids and Strains

The plasmids used in this study are described in Table 2. The integrating expression vector pMV306trpT was created by cloning five copies of the transcriptional terminator trp from pEJ414 (Papavinasasundaram et al., 2001) into pMV306 (Stover et al., 1991) using *Kpn*I + *Xba*I. Cloning of the terminator was confirmed by sequencing. The firefly luciferase (*ffluc*) gene was PCR amplified using primers FFluc_*Eco*RI_F and FFluc_*Sal*I_R (Table 1) containing restriction sites as indicated and using pMV306hsp + FFluc (Andreu et al., 2010) as a template. Primers were designed to only amplify the coding region and hence exclude the optimized Shine–Dalgarno sequence. The sequence of the PCR product was confirmed by DNA sequencing. The *ffluc* gene was cloned into pMV306tprT after digestion with *Eco*RI-*Sal*I to create pMV306trpT-FFluc(_*del*_SD), herein referred as pTC1. The *desA1* (Rv0824c) and *desA2* (Rv1094) gene pair from *M. tuberculosis* were selected as Shine–Dalgarno and leaderless candidates, respectively. Reporter constructs were generated by fusing the candidate regions from *desA1* and *desA2* genes to the *ffluc* gene in pTC1 using *Nco*I-*Eco*RI as follows: (a) the Shine–Dalgarno reporter vector pTC1 + *PdesA1*:*desA1’* was created by fusing the 50 bp promoter region, followed by the 5′UTR and six N-terminal amino acids from *desA1*; (b) the leaderless reporter pTC1 + *PdesA2*:*desA2’* was created by fusing the 50 nt promoter region followed by the six N-terminal amino acids from *desA2*; (c) the Shine–Dalgarno reporter pTC1 + *PdesA2*:*desA1’* was created by swapping the *desA1* promoter in pTC1 + *PdesA1*:*desA1*’ by the *desA2* promoter; and (d) the leaderless pTC1 + *PdesA1*:*desA2*’ reporter was created by swapping the *desA2* promoter in pTC1 + *PdesA2*:*desA2’* by the *desA1* promoter. All candidate regions were introduced by oligo cloning and the sequences of the DNA oligos are available in Table 1. Briefly, oligos were reannealed by mixing equal volumes of both complementary oligos and placing them in a thermocycler with the following settings: first heat at 95°C for 2 min; second cool down to 25°C and incubate for 45 min and finally cool down to 4°C for further storage. All the sequences from the reporter constructs were confirmed by sequencing. Reporter plasmids were transformed into *E. coli*, and electroporated into *M. smegmatis* and *M. tuberculosis* as previously described (Goude et al., 2015). Correct construction of the reporter strains was verified by sequencing of specific PCR amplified regions (Table 1) from either plasmid DNA isolated from *E. coli* or chromosomal DNA isolated from *M. smegmatis* and *M. tuberculosis*. The generated strains were named according to the reporter fusion they carried.

**TABLE 2:** Vectors used in this study.

Vector	Description	Reference or source
pEJ414	pMV306 derivative containing a promoterless *E. coli lacZ* gene	Papavinasasundaram et al., 2001
pMV306	Mycobacterial integrating vector, Km^r^	Stover et al., 1991
pMV306hsp + FFluc	pMV306 derivative containing P*_*hsp*__60_* and the firefly luciferase (FFluc) codon optimized for *M. tuberculosis*	Andreu et al., 2010
pMV306trpT	pMV306 derivative containing 5 copies of transcriptional terminator from pEJ414	This study
pTC1	pMV306trpT derivative encoding the firefly luciferase gene (FFluc) excluding the SD sequence	This study
pTC1 + *PdesA1*:*desA1*′	pTC1 derivative containing P*_*desA*__1_*:UTR*_*desA*__1_*:6 N-terminal aa*_*desA*__1_*	This study
pTC1 + *PdesA2*:*desA2*′	pTC1 derivative containing P*_*desA*__2_*:6 N-terminal aa*_*desA*__2_*	This study
pTC1 + *PdesA2*:*desA1*′	pTC1 derivative containing P*_*desA*__2_*:UTR*_*desA*__1_*:6 N-terminal aa*_*desA*__1_*	This study
pTC1 + *PdesA1*:*desA2*′	pTC1 derivative containing P*_*desA*__1_*:6 N-terminal aa*_*desA*__2_*	This study

### RNA Isolation and Determination of Transcription Start Sites (TSSs)

Samples from *M. tuberculosis* pTC1 + *PdesA1*:*desA1*’, pTC1 + *PdesA2*:*desA2*’, pTC1 + *PdesA2*:*desA1’* and pTC1 + *PdesA1*:*desA2’* reporter strains were harvested from mid-exponential and stationary phase cultures and immediately processed for RNA extraction. For each sample, 30 mL of culture were spun down and RNA isolated using the FastRNA Pro blue kit (MP Biomedicals) following manufacturer’s instructions. All RNA samples were treated with Turbo DNase (Ambion) to remove any DNA contamination. The concentration and quality of RNA samples were assessed by Nanodrop (ND-1000, Labtech) and by running an Agilent RNA chip (2100 Bioanalyser). One microgram of purified RNA was then reverse transcribed into cDNA using the 5′RACE kit from Invitrogen, according to manufacturer’s instructions, and the obtained cDNA was further amplified using the specific oligo FFluc_R1 (Table 1) together with the oligos provided in the 5′ RACE kit. Amplified products were sequenced and the TSSs for each construct identified.

### Luciferase Activity Assays

**FIGURE 1:**
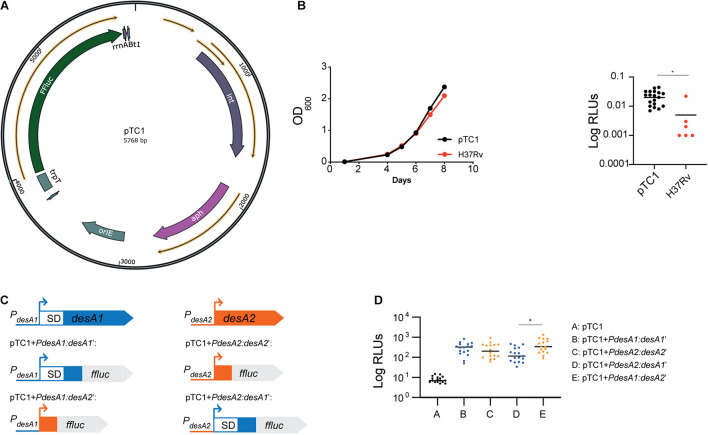
Construction of leaderless and Shine–Dalgarno luminescent reporter strains in *M. tuberculosis*. **(A)** Circular map of plasmid pTC1 [pMV306trpT-FFluc(_*del*_SD)]. The transcriptional terminator and firefly luciferase gene are designated as trpT and FFluc respectively. For construction of downstream plasmids, the required regulatory regions were cloned between these two regions. Circular map was generated with the SnapGene Software (from Insightful Science; available at snapgene.com). **(B)** Bioluminescence production of *M. tuberculosis* carrying an integrated copy of pTC1. Integration of pTC1 in the *M. tuberculosis* chromosome (black) does not affect the growth (measured as OD_600_) when compared to wild-type *M. tuberculosis* (red). Median bioluminescence production (measured as relative light units, RLUs) in the pTC1 *M. tuberculosis* strain (black) was higher to that in the H37Rv wild-type strain with no *FFluc* gene (red). Asterisk denote significant differences (Unpaired *t*-test, *p* = 0.01). Horizontal lines represent median levels of luminescence. **(C)** Schematic representation of the translational reporters constructed using the *M. tuberculosis desA1* and *desA2* genes. All reporters were constructed using the pTC1 vector as backbone. Promoter regions of *desA1* and *desA2* are represented as blue and orange lines, respectively, and the sequence encoding the first 6 amino acids of *desA1* and *desA2* are represented as blue and orange boxes, respectively. For *desA1*, the 5′UTR including the Shine–Dalgarno (SD) sequence is represented as a white box with blue borders. **(D)** Bioluminescence in *M. tuberculosis* transformed with the reporter vectors. For each reporter, luminescence of two independent *M. tuberculosis* transformants was measured daily over 5 days of exponential growth. Results are given as relative light units (RLUs) and are corrected by the optical density. Each dot represents one measurement and bars indicate median values. Asterisk denotes significant differences between pTC1 + *PdesA2*:*desA1*’ and pTC1 + *PdesA1*:*desA2*’ reporter strains (Kruskal–Wallis test, p = 0.009).

To assess the translation kinetics of the leaderless and SD reporters, luciferase activity assays were performed. The substrate for FFluc, D-luciferin (GoldBio^®^), was prepared in distilled water at 94 mM (30 mg ml^–1^) and filter sterilized. All stocks were stored at −20°C and diluted in equivalent broth media or PBS (without calcium or magnesium) immediately before use. Working solutions were kept on ice in the dark during preparation. For the experiments, two or three independent cultures of each strain were grown as described in the bacterial strains and growth conditions section within Materials and methods, and each culture was measured in duplicate or triplicate. Data presented in Figure 1B was acquired using a TD 20/20 tube luminometer (Turner Designs) using 500 μl of sample and 500 μl of D-luciferin solution (final concentration of 150 μg ml^–1^) and default settings. All other data was acquired at 37°C with a FLUOstar Omega microplate reader (BMG Labtech). For measuring luminescence during the THP-1 infection experiments, infected macrophages were washed twice with Hank’s Balanced Salt Solution (HBSS) (ThermoFisher Scientific UK Ltd) to eliminate any extracellular bacteria and subsequently lysed using 0.1% Triton X-100 in HBSS and luminescence from released intracellular bacteria was measured. For performing the measurements, 96-well polystyrene microplates were prepared with 100 μl sample/well, and 10 μl of D-luciferin solution (final concentration of 150 μg ml^–1^) was injected row by row and luminescence was immediately measured after adding the substrate for 3 s. Equivalent blank samples only containing broth medium were processed in parallel to each experiment and the luminescence measurements treated as the background luminescence. These control values were equivalent to luminescence measurements obtained for adequately processed cultures of *M. tuberculosis, M. smegmatis* and *E. coli* carrying the corresponding empty vector pTC1. Data was analyzed using the Mars software package (BMG Labtech) to calculate relative light units (RLUs).

### Nutrient Starvation Experiments

For the nutrient starvation experiments, *M. tuberculosis* H37Rv was grown in 100 ml cultures until mid-exponential phase using 1 L roller bottles (Nalgene). When the cultures reached an OD_600_ of 0.4–0.6, cells were harvested, washed twice with PBS and resuspended in 100 ml of fresh PBS supplemented with 0.025% tyloxapol (Sigma). Cultures were then incubated in 1 L roller bottles for 28 days. Parallel cultures were kept in 7H9 media as controls. For each reporter tested, two independent transformants were grown. The samples were collected from nutrient-starved and control cultures over 28 days for luminescence assays and for RNA extraction. Experiments were performed in duplicate.

### Nitric Oxide Experiments

For the nitric oxide experiments, *M. tuberculosis* H37Rv was grown in 100 ml cultures until mid-exponential phase using 1 L roller bottles. Diethylenetriamine/nitric oxide adduct (DETA/NO; Sigma) was prepared by dissolving in 0.1 M NaOH to reach a working concentration of 100 mM. When the cultures reached an OD_600_ of 0.4–0.6, DETA/NO was added to a final concentration of 0.25 mM. Cultures were subsequently incubated in 1 L roller bottles for 7 days in parallel to control cultures kept in 7H9 with an equivalent concentration of NaOH to that of the DETA/NO cultures. For each reporter tested, two independent transformants were included. Samples were collected after 6, 24, 48, 72, 96, and 120 h for luminescence assays and for RNA extraction. Experiments were performed in duplicate.

### THP-1 Cell Line Culture and Macrophage Infection Experiments

THP-1 line monocytes (ATCC^®^) were cultured routinely in RPMI-1640 medium containing HEPES and L-Glutamine (ThermoFisher Scientific UK Ltd), supplemented into RPMI complete medium with 10% heat-inactivated fetal bovine serum (FBS; Sigma) and 0.05 mM 2-mercaptoethanol (Sigma) at 37°C in 5% CO_2_. The monocytes were differentiated into THP-1 macrophages by using the inducing reagent phorbol 12-myristate 13-acetate (PMA; Sigma) at a final concentration of 0.050 μg ml^–1^. Cells were seeded in 24-well plates at a density of 10^4^ cells/well in 500 μL of medium and differentiated for 4 days prior to infection with *M. tuberculosis*. Mid-log phase cultures of *M. tuberculosis* reporter strains were washed twice with PBS, diluted in RPMI-1640 and added to THP-1 macrophage layers at a concentration of 10^5^ CFU/well (MOI of 10). After 3 h of infection at 37°C in 5% CO_2_, macrophages were washed twice with Hank’s Balanced Salt Solution (HBSS) (ThermoFisher Scientific UK Ltd) to eliminate any extracellular bacteria. Lastly, 0.5 ml of complete RPMI was added to each well. Cells were either immediately processed or incubated further to reach 24, 48, 72, 96, and 168 h (7 days) post-infection. Bacterial intracellular survival and growth were assessed by lysis of the monolayers after addition of 0.1% Triton X-100 in HBSS and enumeration of bacteria by plating of serial dilutions in PBS-Tween onto 7H11 medium. Colonies were counted after 3–4 weeks and their count confirmed after 6 weeks of incubation at 37°C and the average CFU/ml determined. Inoculum represents the CFU/ml of the bacterial population used for the infections. Infection experiments were conducted in triplicate.

### RNA Isolation and q-RT PCR

For the quantification of transcript levels through quantitative real-time PCR, parallel samples to the ones used for the luminescence screens were harvested for RNA isolation. For each sample, 10 mL of culture were spun down and RNA isolated using the FastRNA Pro blue kit (MP Biomedicals), following manufacturer’s instructions. All RNA samples were treated with Turbo DNase (Ambion) to remove any DNA contamination. The concentration and quality of the RNA samples were assessed by Nanodrop (ND-1000, Labtech) and by running an Agilent RNA chip (2100 Bioanalyser). Either 500 ng or 1 μg of the purified RNA were used for reverse transcription into cDNA using the High-Capacity cDNA Reverse Transcription kit (Applied Biosystems). Quantitative real-time PCR was carried out on a 7500 Fast Real-Time PCR system (Applied Biosystems) and using the KAPA SYBR FAST kit (Applied Biosystems), following the manufacturer’s protocol. Specific primers targeting the 16s rRNA were used as an (Table 1) internal control. Relative quantification of the expression levels was calculated using the 2^–ΔΔ*CT*^ method (Livak and Schmittgen, 2001).

### Statistical Analysis

Statistical analysis was performed using GraphPad Prism 9.0.1 (GraphPad Software, San Diego, CA, United States^2^). Data was tested for normal distribution using the D’Agostino & Pearson omnibus test. Differences in translation efficiencies between reporters were assessed using unpaired multiple *t*-tests with False Discovery Rate correction when comparing two groups, or by one-way ANOVA with Tukey’s correction for multiple testing when comparing more than two groups. Non-parametric analysis was performed using the Mann–Whitney test or the Kruskal–Wallis test when comparing two or more groups respectively.

## Results

### Construction of Shine–Dalgarno and Leaderless *M. tuberculosis* Reporter Strains

To study the role of leaderless translation in *M. tuberculosis* response to stress and infection, we first devised a system to easily quantify translation in *M. tuberculosis* by engineering a promoterless firefly luciferase (FFluc) encoding vector that could be used to generate suitable integrative translational reporters. As backbone we used the mycobacterial integrating vector pMV306 (Stover et al., 1991) with five copies of the transcriptional terminator *trp* (Papavinasasundaram et al., 2001). We then cloned the *ffluc* coding region codon-optimized for *M. tuberculosis* from pMV306hsp + FFluc (Andreu et al., 2010) and removed the optimized Shine–Dalgarno sequence to create pMV306trpT-FFluc(_*del*_SD), which we refer herein to as pTC1 (Figure 1A). Median bioluminescence production in the resulting strain was significantly increased to that in the H37Rv wild-type strain with no *ffluc* gene (unpaired *t*-test, *p* = 0.01), indicating that there was some background transcription and translation of the *ffluc* gene in the promoterless vector (Figure 1B).

To compare translational efficiency between leaderless and Shine–Dalgarno transcripts, we selected the *desA1* (Rv0824c) and *desA2* (Rv1094) gene pair which encode homologous acyl-ACP desaturases sharing 30% primary sequence identity. Both genes are strongly expressed during exponential growth and have a typical TANNNT -10 promoter motif; however, while *desA1* contains a 5′UTR with the Shine–Dalgarno sequence, *desA2* is expressed as a leaderless transcript (Cortes et al., 2013). We obtained the Shine–Dalgarno reporter construct pTC1 + *PdesA1*:*desA1’* by fusing the *desA1* 50 bp promoter region, the 5′UTR and the region encoding the first six amino acids of *desA1* to *ffluc* in pTC1 (Figure 1C); similarly, we generated the leaderless reporter construct pTC1 + *PdesA2*:*desA2’* by fusing the *desA2* 50 bp promoter region and the region encoding the first six amino acids of *desA2 to ffluc* in pTC1 (Figure 1C). To make sure that any differences observed between the Shine–Dalgarno and leaderless reporters were due to translational regulation and not to transcriptional regulation, we swapped the 50-bp *desA1* and *desA2* promoters in pTC1 + *PdesA1*:*desA1’* and pTC1 + *PdesA2*:*desA2’* to create the Shine–Dalgarno reporter pTC1 + *PdesA2*:*desA1’* and the leaderless reporter pTC1 + *PdesA1*:*desA2’* respectively (Figure 1C). All reporter sequences were verified by sequencing and plasmids were subsequently electroporated in *M. tuberculosis.* Successful integration was verified by polymerase chain reaction (PCR) followed by sequencing. Additionally, we verified that transcription of the reporters was driven by the desired promoter by determining the transcriptional start sites using 5′ rapid amplification of cDNA ends (RACE). Finally, we verified translation of the FFluc reporter by measuring luminescence of the reporter strains over a 5-day period of exponential growth as an indirect measurement of protein expression. All reporter strains pairs showed comparable levels of luminescence (Kruskal–Wallis test) (Figure 1D) suggesting comparable levels of luciferase production. As a result, we selected the Shine–Dalgarno pTC1 + *PdesA1*:*desA1’* and leaderless pTC1 + *PdesA1*:*desA2’* reporter pair, for which pTC1 + *PdesA1*:*desA1’* contains a naturally occurring canonical Shine–Dalgarno organization for further experiments.

### The Leaderless Reporter Is Robustly Translated in *M. tuberculosis* During *in vitro* Growth

Our results so far suggested that the leaderless reporter was translated with similar efficiency to that of the Shine–Dalgarno reporter at least during exponential growth (Figure 1D). To identify possible differences in the translation efficiencies of the Shine–Dalgarno and leaderless reporters that could be associated with the growth status of the bacteria we used pTC1 + *PdesA1*:*desA1’* and pTC1 + *PdesA1*:*desA2’* to closely monitor luminescence production during the transition from exponential to non-exponential growth. As a control, we also measured luminescence production of the reporters in *M. smegmatis*, a close relative of *M. tuberculosis* that also encodes a high percentage of leaderless proteins in its genome (Shell et al., 2015). Previous experiments using fluorescent reporters have shown that leaderless translation in *M. smegmatis* has similar efficiency to that of a Shine–Dalgarno reporter during exponential growth conditions (Nguyen et al., 2020). Additionally, we measured luminescence production of the reporters in *E. coli*, a bacterium with scarce leaderless transcripts that are translated at low levels by the 70S monosome (Moll et al., 2002).

**FIGURE 2:**
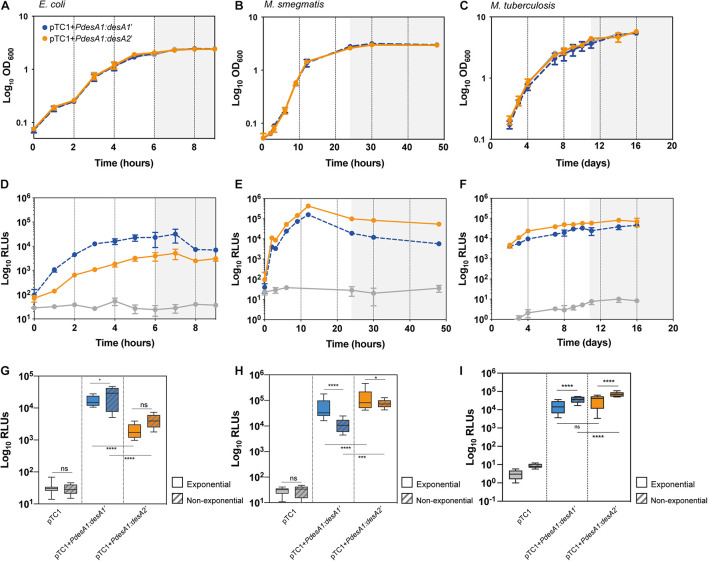
Growth and translation kinetics of leaderless and Shine–Dalgarno reporters in three bacterial models during *in vitro* growth. Bacterial growth curves of *E. coli* **(A)**, *M. smegmatis* **(B)** and *M. tuberculosis* **(C)** and luminescence measured in *E. coli* **(D)**, *M. smegmatis* **(E)** and *M. tuberculosis* **(F)** transformed with pTC1 (gray), pTC1 + *PdesA1*:*desA1*’ (Shine–Dalgarno, blue) and pTC1 + *PdesA1*:*desA2’* (leaderless, orange). The vectors are replicative in *E. coli* and integrative in mycobacteria. Cultures were inoculated to an initial OD ∼ 0.1 and growth (expressed as OD_600_) and luminescence (expressed as relative light units, RLUs) were measured over 9 h for *E. coli* **(A,D)**, 48 h for *M. smegmatis* **(B,E)** and 16 days for *M. tuberculosis* **(C,F)**. Gray background within panels **(A–F)** indicates non-exponential growth. For each timepoint and reporter at least three independent transformants were analyzed and each experiment was performed in triplicate. The mean value and standard deviation are presented. Luminescence levels during exponential growth (clear boxes) and non-exponential growth (patterned boxes) in *E. coli* **(G)**, *M. smegmatis* **(H),** and *M. tuberculosis* **(I)** transformed with the indicated vectors. Box plots indicate median (horizontal line), interquartile range (box) and maximum and minimum values (whiskers). For *E. coli* **(G)**, exponential growth was considered for time points from 2 to 5 h and non-exponential growth (stationary growth) from 6 to 9 h (highlighted with gray background in **A,D**). For *M. smegmatis* **(H)**, the exponential phase of growth was defined for time points from 6 to 12 h and non-exponential growth (stationary) for time points 24–48 h (highlighted with gray background in **B,E**). For *M. tuberculosis* **(I)**, exponential growth phase was considered for time points 2–10 days and non-exponential growth from 11 days (highlighted with gray background in C,F). Means were compared using one-way ANOVA. *p < 0.05, ***p < 0.001, ****p < 0.0001, ns, non-significant.

Introduction of the reporter vectors did not affect bacterial growth in any of the three bacterial models tested (Figures 2A–C). As predicted, during exponential growth luminescence production from the leaderless reporter in *E. coli* was 10-fold lower than that from the Shine–Dalgarno reporter (one-way ANOVA, *p* < 0.0001; Figures 2D,G and Supplementary Figure 1A). By contrast, in *M. smegmatis*, luminescence from the leaderless reporter was significantly higher than that from the Shine–Dalgarno reporter (one-way ANOVA, *p* < 0.001) (Figures 2E,H and Supplementary Figure 1A), whereas in *M. tuberculosis* luminescence production of the leaderless reporter was comparable to that of the Shine–Dalgarno reporter (Figures 2F,I and Supplementary Figure 1A). Upon exit from exponential growth, median luminescence levels increased for both reporters in *E. coli*, but the increase was not statistically significant in the case of the leaderless reporter (Figure 2G). In *M. smegmatis*, the mean luminescence levels of the Shine–Dalgarno reporter significantly decreased whilst the mean luminescence levels of the leaderless reporter were maintained (one-way ANOVA, *p* < 0.0001) (Figure 2H). In the case of *M. tuberculosis*, both Shine–Dalgarno and leaderless translation were significantly increased upon exit from exponential growth (one-way ANOVA, *p* < 0.0001) (Figure 2I).

In summary, our results show a robust translation of the leaderless reporter during exponential growth in *M. tuberculosis*, to a level comparable to that of the Shine–Dalgarno reporter. Upon exit from exponential growth, the mean levels of both reporters significantly increased compared to those during exponential growth, but mean levels of leaderless translation were significantly higher than mean levels of Shine–Dalgarno translation.

### The Preference for Translation of the Leaderless Reporter Varies During Different *in vitro* Stresses in *M. tuberculosis*

Despite mean levels of leaderless translation being significantly higher than mean levels of Shine–Dalgarno translation upon exit from exponential growth, our results did not indicate that the ratio of translation of leaderless and Shine–Dalgarno transcripts was different during the transition from exponential to non-exponential growth in *M. tuberculosis* (Supplementary Figure 1B). Next, we studied what happens during stress conditions. In particular, we determined the luminescence production of leaderless and Shine–Dalgarno reporters in *M. tuberculosis* during nutrient starvation and following a transient stress with a sub-lethal concentration of NO. These two conditions are representative of conditions encountered by *M. tuberculosis* during non-replicating growth (Betts et al., 2002) and active infection in humans (Nathan, 2006; Robinson et al., 2014), respectively. In each condition tested, we addressed translation kinetics of the leaderless and Shine–Dalgarno reporters by measuring luminescence produced from the pTC1 + *PdesA1*:*desA1’* and pTC1 + *PdesA1*:*desA2’* reporter vectors and quantified changes at the transcriptional level through quantitative real-time PCR to rule out changes due to differences in promoter activities.

**FIGURE 3:**
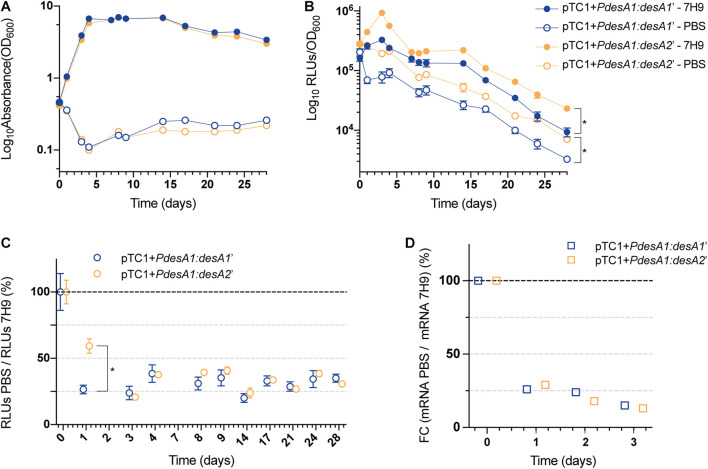
Growth and translation kinetics of the leaderless and Shine–Dalgarno reporters during nutrient starvation. Cultures of *M. tuberculosis* carrying integrative vectors pTC1 + *PdesA1*:*desA1’* [Shine–Dalgarno (SD), blue] and pTC1 + *PdesA1*:*desA2*’ [leaderless (L), orange] were grown to mid-exponential phase (time 0), washed and resuspended in PBS or kept in rich media as controls (7H9). For each timepoint and reporter at least three independent transformants were used and measurements were done in triplicate; the mean value and standard deviation are presented. **(A)** OD_600_ was monitored for 28 days. **(B)** Luminescence, given as relative light units (RLUs) corrected by growth. Asterisks denote statistically significant differences between Shine–Dalgarno and leaderless reporters for all timepoints in each condition (7H9 and PBS) (multiple *t*-tests, *p* < 0.001). **(C)** To calculate the overall effect that nutrient starvation has on the translation of the reporters, for each timepoint and reporter, the RLU value during nutrient starvation (PBS) was normalized against the RLU control value (7H9). The value obtained at time 0 was considered as the 100% translation efficiency. Statistically significant differences are indicated with an asterisk (multiple *t*-tests, *p* < 0.001). **(D)** Quantification of mRNA levels by real-time PCR. 16S rRNA was used as the internal control. Fold changes (FC) are relative to mRNA levels in control cultures (7H9) and the value obtained at time 0 was considered as 100% transcription level.

For the nutrient starvation experiments, cells growing exponentially in nutrient-rich media were washed with PBS and used as the inoculum (time zero). PBS (nutrient starvation) and nutrient-rich media (7H9 media, control) cultures were incubated for 28 days. The control cultures grew exponentially for 5 days before reaching stationary phase (Figure 3A). The control cultures reached stationary phase earlier than the cultures in Figure 2C, likely because of the higher starting OD (see section “Materials and Methods”). As expected, nutrient limitation affected the growth and luminescence production of both the leaderless and Shine–Dalgarno *M. tuberculosis* reporter strains compared to those in the control cultures (Figures 3A,B). In both the control and the nutrient starvation cultures, the leaderless reporter was translated at a higher level than the Shine–Dalgarno reporter (multiple *t*-tests, *p* < 0.03) (Figure 3B and Supplementary Figure 2). When comparing changes in the luminescence production of the reporters during nutrient starvation with those growing in control conditions, we found a significantly higher increase in luminescence production of the leaderless reporter compared to that of the Shine–Dalgarno reporter after 24 h of nutrient starvation (multiple *t*-tests, *p* < 0.001) (Figure 3C). This difference was not driven by transcriptional changes, as transcription levels of the Shine-Dalgarno and leaderless reporters did not change significantly (one-way ANOVA, *p* > 0.163) (Figure 3D). This less pronounced effect of nutrient starvation on leaderless-mediated luminescence production was transient; a comparably high reduction of luminescence production of both types of reporters was observed at 72 h post-nutrient starvation and later time points (Figure 3C).

**FIGURE 4:**
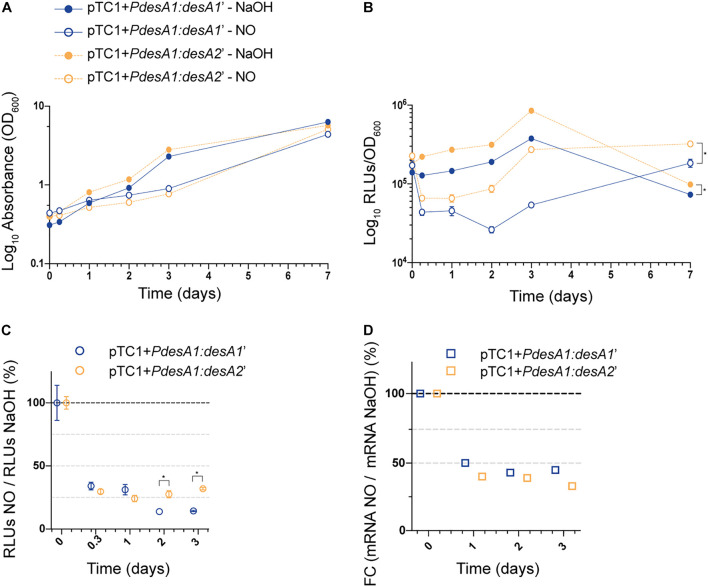
Growth and translation kinetics of the leaderless and Shine–Dalgarno reporters during nitric oxide (NO) stress. Cultures of *M. tuberculosis* carrying integrative vectors pTC1 + *PdesA1*:*desA1’* [Shine-Dalgarno (SD), blue] and pTC1 + *PdesA1*:*desA2*’ [leaderless (L), orange] were grown to mid-exponential phase (time 0) when cells were either challenged with 0.25 mM NO or the vehicle NaOH as control. For each timepoint and reporter at least three independent transformants were used and measurements were done in triplicate; the mean and standard deviation are presented. **(A)** OD_600_ was monitored for 7 days. **(B)** Luminescence, given as relative light units (RLUs) corrected by growth. Asterisks denote statistically significant differences between pTC1 + *PdesA1*:*desA1’* and pTC1 + *PdesA1*:*desA2*’ reporters for all timepoints in each condition (multiple *t*-tests, *p* < 0.03). **(C)** The RLU value during NO challenge was normalized against the corresponding RLU NaOH value. The value obtained at time 0 was considered as 100% translation efficiency. Statistically significant differences are indicated with an asterisk (multiple *t*-tests, *p* < 0.002). **(D)** Quantification of mRNA levels by real-time PCR. 16S rRNA was used as the internal control. Fold changes (FC) are relative to the NaOH corresponding control and the value obtained at time 0 was considered as 100% transcription level.

Next, we studied changes in translation during exposure to a sub-lethal concentration of NO by monitoring changes in luminescence in exponentially growing cultures of the *M. tuberculosis* leaderless and Shine–Dalgarno reporter strains exposed to 0.25 mM NO or sodium hydroxide (NaOH, used to dissolve the NO adduct, see section “Materials and Methods”) for 7 days. We chose 0.25 mM NO as it has been previously demonstrated that exposure to intermediate levels of NO (0.5 and 1.0 mM) results in a temporary cessation of growth (Voskuil et al., 2011). As expected, exposure to NO caused a rapid but transient growth arrest of both the Shine–Dalgarno and leaderless reporter strains that could be observed from 48 h post-NO exposure (Figure 4A). Throughout the experiment, the leaderless reporter was translated at a higher level than the Shine–Dalgarno reporter in both the NO-treated and the NaOH control cultures (multiple *t*-tests, *p* < 0.03) (Figure 4B). Indeed, although the transient inhibition in growth resulted in a decrease in luminescence production of both reporters compared with the corresponding controls, both reporters were affected to a similar degree and hence translation of the leaderless reporter was higher than that of the Shine–Dalgarno reporter (Figures 4B,C). As DETA/NO is reported to release NO with a half-life of 5.5 h under similar *M. tuberculosis* growth conditions (Voskuil et al., 2003), it is likely the effects here observed are due to the initial NO exposure alone. Forty-eight hours after NO treatment started, both reporter strains resumed growth and luminescence production, suggesting that they had recovered from the stress (Figures 4A,B). At this point, the percentage of luminescence production of the leaderless reporter in comparison with that in the corresponding control was significantly higher than that of the Shine-Dalgarno reporter (27% vs. 14%, respectively, multiple *t*-tests, *p* < 0.002), suggesting a quicker recovery of leaderless translation following exposure to NO treatment (Figure 4C). These differences were not driven by transcriptional changes, as we found no significant differences in the transcription levels of Shine–Dalgarno and leaderless transcripts (Tukey’s multiple comparisons test, *p* > 0.162) (Figure 4D).

Overall, the results show that luminescence production of the leaderless reporter is significantly less affected by nutrient starvation and recovers more quickly from nitrosative stress than luminescence production from the Shine-Dalgarno reporter.

### Translation of the Leaderless Reporter Is Less Affected During the Early Stages of Macrophage Infection

Finally, we studied luminescence production of the leaderless and Shine–Dalgarno reporters during *M. tuberculosis* intracellular growth in macrophages. To this end we measured luminescence of the reporter strains during infection of PMA-activated THP-1 cells. We used an MOI of 10 to ensure sufficient intracellular bacteria would be present to allow detection of reporter luminescence.

**FIGURE 5:**
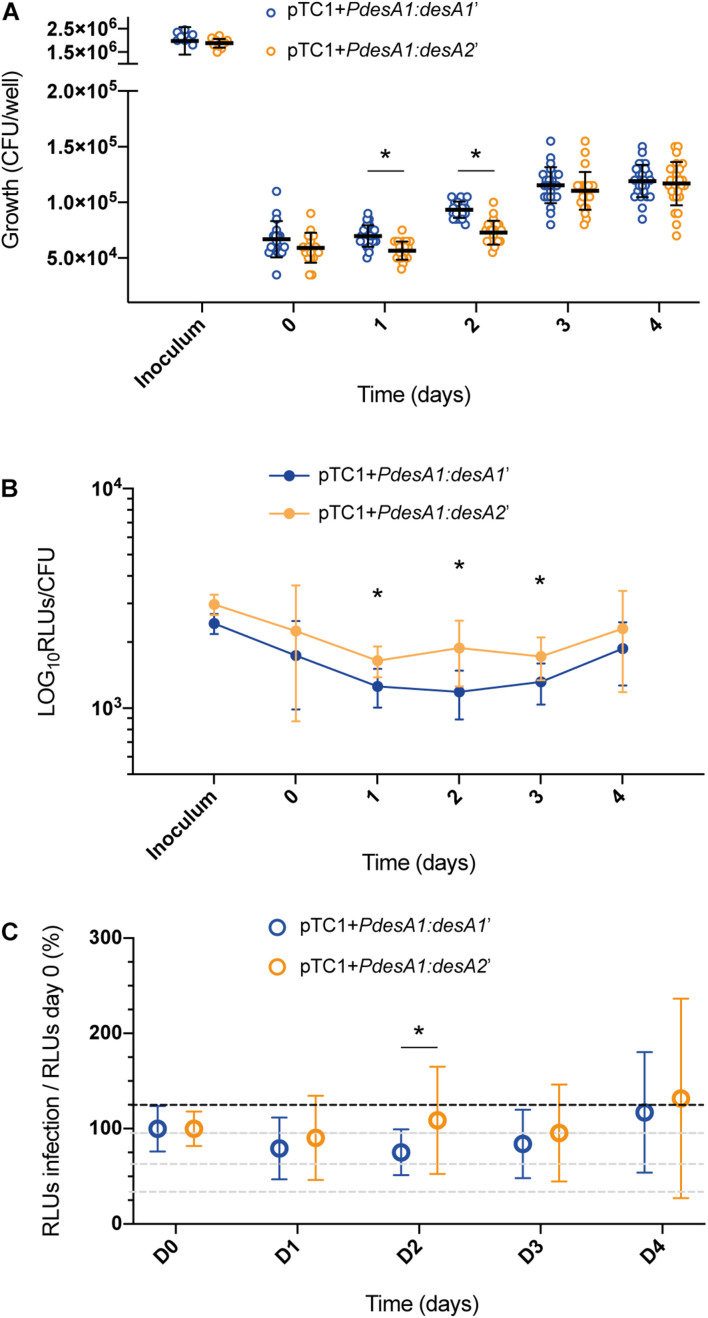
Growth and luminescence production of the leaderless and Shine–Dalgarno reporter strains during infection of THP-1 cells. **(A)** CFU were counted for 4 days post-infection of THP-1 cells with the *M. tuberculosis* leaderless (orange) and Shine–Dalgarno (blue) reporter strains. Inoculum represents the CFU/ml used for the infections. Asterisks denote statistically significant differences between the leaderless and Shine–Dalgarno reporter strains (multiple *t*-tests, *p* < 0.001). **(B)** Luminescence, given as relative light units (RLUs) corrected by CFU. Statistically significant differences at day 1, day 2, and day 3 post infection are indicated with an asterisk (multiple *t*-tests, *p* < 0.002). **(C)** To calculate the overall effect that infection of THP-1 cells has on the translation of the reporters, for each timepoint and reporter, the RLU value during macrophage infection was normalized against the RLU after bacterial internalization (time 0). The value obtained at time 0 was considered as the 100% translation efficiency. All experiments were performed in triplicate. Asterisk denotes significant differences (multiple *t*-test, *p* < 0.05).

Following infection of THP-1 cells, no increase in luminescence and growth was observed for the reporter strains during the first 24 h (Figures 5A,B). At 48 h post infection, both reporter strains resumed growth and translation, suggesting that they had adapted to the intracellular environment. At 24 and 48 h post infection, there was a significant decrease in the growth of the leaderless reporter compared to that of the Shine–Dalgarno reporter (Figure 5A; multiple *t*-tests, *p* < 0.0001), but this reduction on viable bacteria for the leaderless reporter did not correlate with a significant reduction in luminescence production (Supplementary Figure 3A). When corrected by growth, luminescence from the leaderless reporter was higher than that from the Shine–Dalgarno reporter and this difference was significant between 24 and 72 h post infection (multiple *t*-tests, *p* < 0.002) (Figure 5B). To quantify how translation of the leaderless and Shine–Dalgarno reporters responded during the different stages of macrophage infection, we normalized the luminescence levels of the reporters at different time points against the luminescence levels at time 0. This revealed that during the first 48 h of infection, before bacterial growth resumed, translation of the leaderless reporter was less affected than that of the Shine–Dalgarno reporter, and this difference was statistically significant at 48 h post infection (multiple *t*-test, *p* < 0.05) (Figure 5C). As growth resumed following adaptation to the intracellular environment, the luminescence production of both types of transcripts increased to comparable levels (Figure 5C and Supplementary Figure 3B). Our data show that during the early stages of macrophage infection, the luminescence production of the leaderless reporter is significantly less affected by exposure to the intracellular host environment than that of the Shine–Dalgarno.

## Discussion

Adaptation of *M. tuberculosis* to stress conditions by regulation of gene and protein expression is key for survival of this pathogen in the hostile intracellular environment. In this study we aimed to better understand the role of leaderless translation in the response of *M. tuberculosis* to *in vitro* stress and during infection. Our results indicate that luminescence production of the leaderless reporter is more efficient during adaptation to stress conditions in *M. tuberculosis.* Luminescence production of the leaderless reporter was less affected than that of the Shine–Dalgarno reporter upon entrance into nutrient starvation and during the recovery phase from nitrosative stress, as well as during the early stages of macrophage infection.

Transcription of leaderless transcripts has been mapped during nutrient starvation-induced growth arrest in *M. tuberculosis* to show their median abundance significantly increases upon growth arrest (Cortes et al., 2013). Amongst leaderless transcripts significantly upregulated are *M. tuberculosis* stress genes regulated by the “feast to famine” LrpA protein (Rv3291c), alternative sigma factor E (*sigE*, Rv1221), genes from the methylcitrate cycle (Rv1130-Rv1131) and lysine ε-aminotransferase (l*at*, Rv3290c). In *E. coli*, leaderless transcripts are preferentially translated *in vitro* during unfavorable conditions such as exposure to kasugamycin (Kaberdina et al., 2009) or activation of the toxin MazF (Vesper et al., 2011). We selected nutrient starvation, exposure to nitric oxide and infection of macrophages to further study their effect on the translation levels of our leaderless and Shine–Dalgarno reporters. Although ribosome profiling analysis has not revealed an overall difference in the translation efficiency of leaderless transcripts compared to that of Shine–Dalgarno transcripts (Sawyer et al., 2021), our results show that translation of the leaderless reporter, measured as luminescence production, is less affected than that of its matched Shine–Dalgarno reporter. However, this effect is mostly transient, as shown by leaderless and Shine–Dalgarno reporters reaching comparable levels of luminescence after the initial phases of nutrient starvation-induced growth arrest and growth in THP-1 cells. These results suggest that leaderless translation could offer an advantage to Shine–Dalgarno translation under some growth conditions, including starvation-induced growth arrest, even if it would mean just a faster adaptation to environmental changes.

Translation of the leaderless reporter was robust in our system, confirming what has been shown at genome-wide level in both *M. smegmatis* and *M. tuberculosis* (Shell et al., 2015; Sawyer et al., 2021), and highlighting an important difference with the *E. coli* model, where leaderless translation is performed with low efficiency by the *E. coli* ribosome (Moll et al., 2002, 2004; Moll and Engelberg-Kulka, 2012). In fact, introduction of our leaderless reporter into *E. coli* cells showed a 10-fold reduction in luminescence levels compared to that of the Shine–Dalgarno reporter, highlighting mechanistic differences between the two bacterial models. The recently solved crystal structures of the *M. tuberculosis* and *M. smegmatis* ribosomes have revealed two novel ribosomal proteins and a significant degree of structural heterogeneity (Hentschel et al., 2017; Yang et al., 2017), suggesting that either mycobacterial ribosomes have a greater capacity to efficiently translate different transcript organizations or the existence of subpopulations of ribosomes with the ability to preferentially translate different transcripts. In this respect, the identification of at least four additional ribosomal proteins, which can be alternatively incorporated into the ribosome in response to zinc starvation in *M. smegmatis* (Prisic et al., 2015; Dow and Prisic, 2018; Chen et al., 2020), and the identification of ribosome-associated proteins under non-optimal growth conditions in both *M. smegmatis* and *M. tuberculosis* (Trauner et al., 2012; Li et al., 2015), contribute to the body of recent literature highlighting further layers of translational regulation in this pathogen. We have detected robust luminescence production of the leaderless and Shine–Dalgarno reporters under different growth conditions in *M. tuberculosis*, this supports the hypothesis that *M. tuberculosis* ribosomes have the capacity to efficiently translate both Shine–Dalgarno and leaderless transcripts as opposed to relying on a translational reprogramming for selective translation. The transcriptome of *M. tuberculosis* contains many genes that can be transcribed from alternative promoters one of them being leaderless (Cortes et al., 2013). In the case of the sigma factor E *sigE* there is a >10-fold increase in transcription during conditions of nutrient starvation, that is solely driven by the leaderless transcript (Cortes et al., 2013). Understanding the mechanistic differences that lead to initiation of translation of leaderless and Shine–Dalgarno transcripts in *M. tuberculosis* is essential to fully understand the role that alternative translation initiation mechanisms could play in the protein synthesis and stress adaptation of *M. tuberculosis.*

We have observed no differences in luminescence production for the leaderless and Shine–Dalgarno reporters during exponential growth in *M. tuberculosis*. A recent study has investigated the impact of leadered and leaderless translation in *M. smegmatis* during exponential growth using fluorescent translational reporters (Nguyen et al., 2020). Briefly, the 5′UTR sequence of sigma factor *sigA* was used as a representative of leadered translation for the construction of the fluorescent reporters, and compared its fluorescence to that of a leaderless reporter (devoid of the 5′UTR sequence) as well as another leadered reporter carrying the 5′UTR from the semi-synthetic promoter *p_*myc*__1_tetO* instead of the *sigA* 5′UTR. They found that the leaderless reporter was significantly less fluorescent than the leadered counterpart carrying the semi-synthetic 5′UTR, suggesting that leaderless transcripts may be translated with less efficiency; but when leaderless fluorescence was compared to that of the reporter carrying the native *sigA* 5′UTR structure, they observed no significant differences in fluorescence levels (Nguyen et al., 2020), similarly to what we report in this study with luminescent reporters. Interestingly, these findings highlight how different 5′UTR conformations, including those without a Shine–Dalgarno sequence, can severely impact translation rates in mycobacteria and needs to be taken into consideration when drawing more general conclusions about gene regulation in *M. tuberculosis*.

We selected the *desA1* and *desA2* gene pair as representatives of the Shine–Dalgarno and leaderless transcripts in *M. tuberculosis* on the basis of their protein shared homology, widespread expression (Cortes et al., 2013) and conserved features within their promoters, like the presence of a -10 TANNNT *sigA* consensus motif (Sachdeva et al., 2010) or the use of ATG as a start codon (Cortes et al., 2013; Newton-Foot and Gey Van Pittius, 2013). Both genes are expressed at a high level in exponential *in vitro* growth (Cortes et al., 2013; Sawyer et al., 2021). RNA levels for both genes have been reported to significantly decrease under conditions of nutrient starvation (Betts et al., 2002; Cortes et al., 2013; Sawyer et al., 2021) but no changes have been reported under conditions of NO stress (Cortes et al., 2017) or infection of THP-1 cells (Fontán et al., 2008). This suggests that transcript levels for both genes are regulated similarly in the stress conditions here studied. We used luminescent reporters as they provide a sensitive and convenient measurement of protein expression in bulk *M. tuberculosis* cultures (Andreu et al., 2010). As we have only used one pair of leaderless and Shine–Dalgarno reporters to quantify differences in luminescence production, future work is needed to study if the changes here reported are representative of the leaderless transcripts as a whole and to identify mechanistic differences in their translation to that of Shine–Dalgarno transcripts in *M. tuberculosis*. The reporter constructs included the first six amino acids of the *desA1* and *desA2* coding sequences as N-terminal fusions to the *ffluc* gene to ensure that the native sequence elements downstream the start codon were included, as for example in *E. coli* the downstream box has been shown to contribute to the efficiency of translation initiation (Sprengart et al., 1996). Although we cannot completely rule out that some of the differences observed in this study could be somehow linked to the design of our constructs, it is unlikely that these six amino acids differentially affect the luciferase function of the resulting proteins particularly since protein fusions of the firefly luciferase have been widely used with negligible effect on protein production and function (Smirnova and Ugarova, 2017).

This work represents the first report where individual luminescent reporter strains have been used to quantify luminescence production of mRNA transcripts with different architectures during different growth conditions in *M. tuberculosis.* Our results show that during the early stages of starvation-induced growth arrest, exposure to NO and growth in THP-1 cells, luminescence production of the leaderless reporter is transiently less affected than that of the Shine–Dalgarno reporter. Altogether, our data may suggest that leaderless translation could offer a transient advantage to Shine–Dalgarno translation during the early stages of adaptation to different growth conditions in *M. tuberculosis*, where reliance on alternative translation initiation mechanisms could be advantageous.

## Data Availability Statement

The original contributions presented in the study are included in the article/Supplementary Material, further inquiries can be directed to the corresponding author/s.

## Author Contributions

NA created the pMV306trpT integrating vector and with TC and AG designed the translational reporters. AG and TC conceived and designed the experiments, analyzed the data, and wrote the manuscript. AG performed the experiments. NA revised the manuscript. All the authors read and approved the final manuscript.

## Conflict of Interest

The authors declare that the research was conducted in the absence of any commercial or financial relationships that could be construed as a potential conflict of interest.

## Publisher’s Note

All claims expressed in this article are solely those of the authors and do not necessarily represent those of their affiliated organizations, or those of the publisher, the editors and the reviewers. Any product that may be evaluated in this article, or claim that may be made by its manufacturer, is not guaranteed or endorsed by the publisher.

## Footnotes

1
https://www.sourcebioscience.com

2
www.graphpad.com
