# Supplementary material for: Translation of a Leaderless Reporter Is Robust During Exponential Growth and Well Sustained During Stress Conditions in Mycobacterium tuberculosis
Source: Front Microbiol. 2021 Sep 17;12:746320. doi: 10.3389/fmicb.2021.746320 (PMC8485053; doi:10.3389/fmicb.2021.746320)
Supplement: Supplementary file 1 [file Data_Sheet_1.pdf]

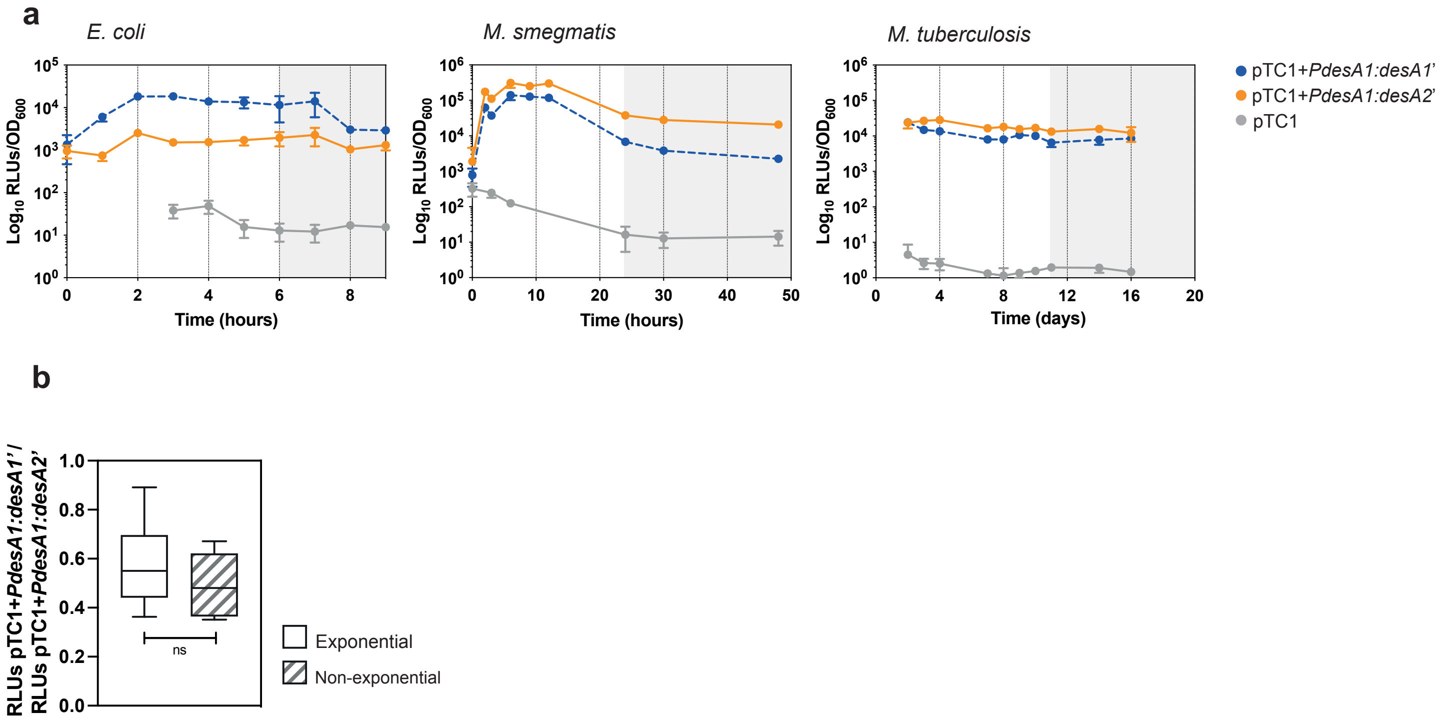

Supplementary Figure 1. a) Luminescence, given as relative light units (RLUs) corrected by growth of leaderless and Shine-Dalgarno reporters in *E. coli* , *M. smegmatis* and *M. tuberculosis* during *in vitro* growth. Strains were transformed with pTC1 (grey), pTC1+PdesA1*:desA1'* (Shine-Dalgarno, blue) and pTC1+PdesA1:desA2' (leaderless, orange). Grey background within panels a-c indicates non-exponential growth. For each timepoint and reporter at least three independent trans-formants were analyzed and each experiment was performed in triplicate. The mean value and standard deviation are presented. b) Ratio of RLUs for the Shine-Dalgarno reporter (pTC1+*PdesA1:desA1')* versus RLUs for the leaderless report-er (pTC1+*PdesA1:desA2')* in *M. tuberculosis* during conditions of exponential growth (clear box) and non-exponential growth (patterned box).

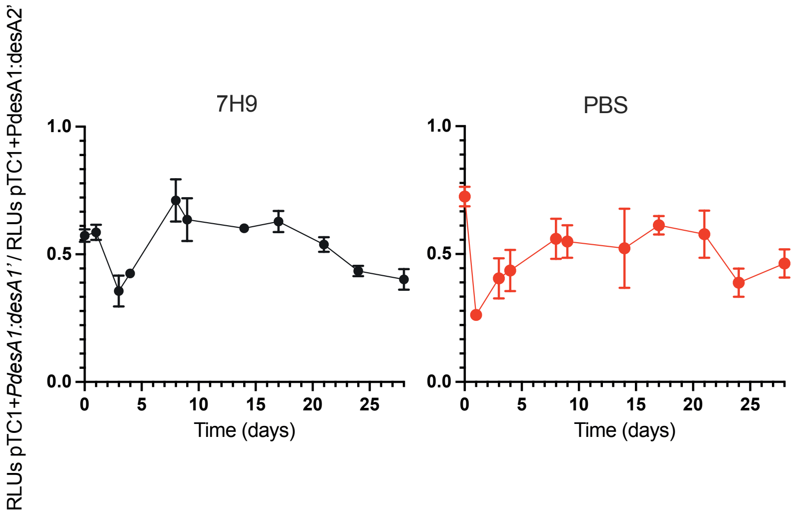

Supplementary Figure 2. Ratio of RLUs for the Shine-Dalgarno reporter (pTC1+*PdesA1:desA1')* versus RLUs for the leaderless reporter (pTC1+*PdesA1:desA2')* in *M. tuberculosis* during conditions of growth in rich media (7H9) and conditions of nutrient starvation (PBS).

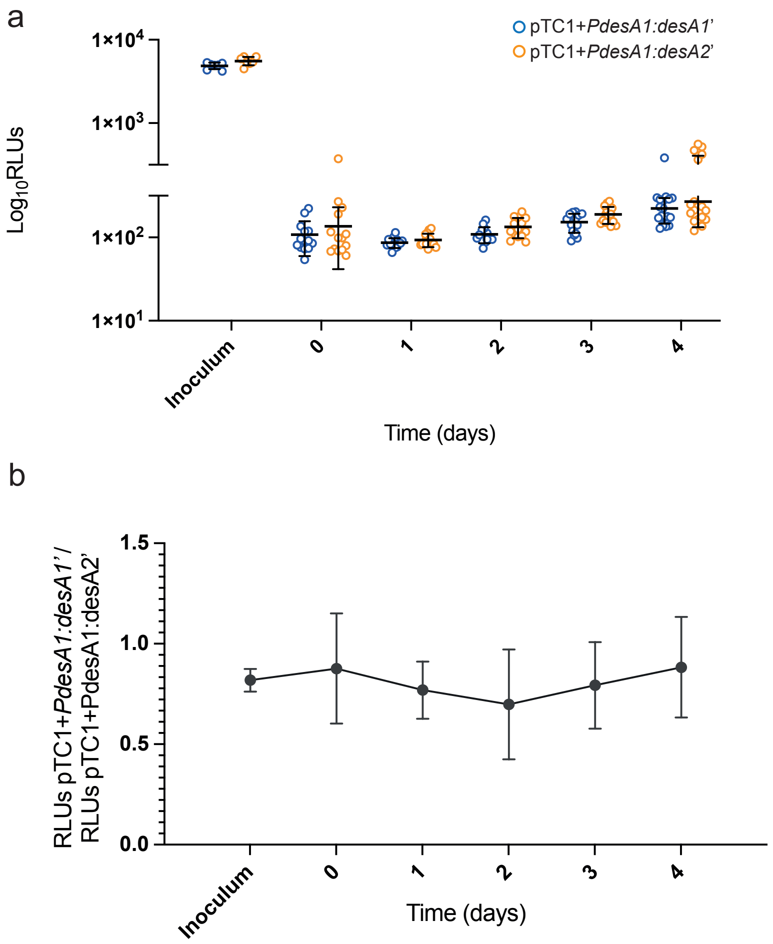

Supplementary Figure 3. **a)** Luminescence production of the leaderless (pTC1+*PdesA1:desA2'*, orange) and Shine-Dalgarno (pTC1+*PdesA1:desA1'*, blue) reporter strains during infection of THP-1 cells. Luminescence is given as relative light units (RLUs). Luminescence was monitored for 4 days post-infection. No significant differences were observed between the Shine-Dalgarno and the leaderless reporter strains at any timepoint (mutiple t-tests). **b)** Ratio of RLUs for the Shine-Dalgar-no reporter (pTC1+*PdesA1:desA1')* versus RLUs for the leaderless reporter (pTC1+*PdesA1:desA2')* in *M. tuberculosis* during infection of THP-1 cells.
